# Factor VIII inhibitor development in Egyptian hemophilia patients: does intron 22 inversion mutation play a role?

**DOI:** 10.1186/s13052-020-00878-5

**Published:** 2020-09-14

**Authors:** Laila M. Sherief, Osama A. Gaber, Hala Mosaad Youssef, Hanan S. Sherbiny, Wesam a Mokhtar, Asmaa A. A. Ali, Naglaa M. Kamal, Yehia H. Abdel Maksoud

**Affiliations:** 1 Pediatric Department, Faculty of Medicine, Zagazig University, Zagazig, Egypt; 2 Biochemistry Department, Faculty of Medicine, Zagazig University, Zagazig, Egypt; 3 Pediatric Department, Collage of Medicine, University of Bisha (UB), Bisha, Kingdom of Saudi Arabia; 4 Pediatric Department, Faculty of Medicine, Cairo University, Cairo, Egypt; 5 Pediatric Department, Faculty of Medicine, Benha University, Benha, Egypt

**Keywords:** Factor VIII inhibitor, Hemophilia, Intron 22 inversion mutation, Children

## Abstract

**Background:**

Hemophilia A (HA) is an X-linked recessive bleeding disorder characterized by qualitative and quantitative deficiency of factor VIII (FVIII). The development of inhibitor antibodies against FVIII is the most challenging complication of treatment. Mutations in the FVIII gene is one of the genetic factors that leads to development of FVIII inhibitors especially intron 22 inversion (Inv22).

**Objectives:**

This study was carried out to assess the frequency of Inv22 of FVIII gene in Egyptian patients with hemophilia A and its role as a risk factor for developing inhibitors.

**Patients and methods:**

Seventy-two patients with severe HA and 48 patients with moderate HA were enrolled in the current study. All patients were treated on demand with either plasma-derived factor VIII or recombinant factor VIII concentrates. Genotyping of FVIII Inv22 was performed by LD-PCR while the presence and magnitude of inhibitor activity in blood was determined by the Bethesda assay.

**Results:**

Around 23% of all hemophilia cases had positive Inv22. Intron 22 inversion mutation was detected in 6 and 33% of patients with moderate and severe HA respectively. Twenty-one cases (18%) of all hemophilic patients developed inhibitors. Thirty-7% of patients with Inv22 had inhibitor in their blood, almost all, but one, had severe HA. The risk of an inhibitor development during replacement therapy was four folds higher among Inv22 positive cases as compared with mutation negative peers (OR 4.3, 95% CI 1.6–11.9, *P* = 0.003).

**Conclusions:**

The prevalence of Inv22 of F VIII in Egyptian hemophiliacs is nearly like that of other population. This mutation was more frequently detected among severe hemophilic patients as compared with moderately affected peers. The presence of Inv22 mutation significantly predispose to FVIII inhibitor development.

## Introduction

Hemophilia A (HA) is the most common X-linked recessive coagulation disorder, affecting 1:5000–7000 male live birth worldwide [1]. It is caused by mutation in factor 8 (FVIII) gene which leads to deficiency of FVIII, a complex glycoprotein which is primarily synthetized by hepatocytes and plays an important role in hemostatic mechanism [2].

Human FVIII gene is located at band 28 on long arm of X-chromosome, having 26 exons and 25 intervening introns [3]. X-chromosome contains three copies of FVIII and its adjacent region, one in intron 22 and two in transcription starting site [4].

FVIII gene defects namely large rearrangements, insertion, and deletion may result in severe disease. However, the single most clinically important mutation is a recurrent large gene rearrangement; an inversion; involving FVIII intron 22 (Inv22) that leads to almost half of all severely affected cases of hemophilia-A globally [3].

The age of diagnosis and frequency of bleeding episodes related to the level of FVIII clotting activity. Hemophilia patients have prolonged partial thromboplastin time (aPTT) with normal prothrombin (PT) and bleeding time. Definitive diagnosis is based on specific assay of FVIII coagulant activity [1] which can be assessed by one-stage clotting assay based on aPTT activity, chromogenic assay for FVIII, or infrequently by immunogenic method [5].

Inhibitor development against FVIII occurs in approximately 20–30% of severe hemophilia patients. It is a multifactorial process involves both genetic and/or environmental factors [6, 7]. Genetic deviation such as Inv22 together with other mutation; nonsense and large deletion; are associated with high risk of developing inhibitors when these patients are treated with FVIII [8]. Environmental factors such as family history of inhibitors, factor replacement therapy types and regimens, intensive FVIII therapy related to surgery or trauma [9] and immunological challenges (i.e. vaccination, viral infection) have been implicated as risks for inhibitor development [7].

Knowledge about the prevalence of different causative mutation of FVIII gene among hemophilia patients in most developing countries, is scarce due to limited resources [10]. The current research was conducted to assess the prevalence of Inv22 mutation of FVIII gene among Egyptian hemophilic patients and its role in inhibitor development during FVIII replacement therapy.

## Patients and methods

### Patients

The current cross-sectional study was carried out at Hematology unit, Pediatric and Biochemistry departments, Zagazig University, Zagazig, Egypt, in the period between April 2018–April 2019. The study was approved by the Institutional Review Board of Faculty of Medicine, Zagazig University.

All patients with moderate and severe hemophilia-A were enrolled in the study. If more than one affected family member, only one out of all affected family members was included. They were treated with either plasma derived FVIII or recombinant FVIII concentrates and occasionally cryoprecipitate as “on demand” regimen according to the availability of different types in our center. No prophylactic regimen is implemented in our institute due to lack of resources.

Mildly affected patients and family-relatives (only one per family was accepted) were excluded, while recently transfused, with FVIII, blood, or other blood product (1 week before), and those with current severe bacterial or viral infection were postponed.

Written informed consents for inclusion in the study were obtained from patients or their legal guardians.

Eligible patients were subjected to full history taking (personal, family and medical), thorough physical examination with particular attention to signs of increased bleeding tendencies and associated complications (hematomas, arthropathy).

Hemophilia severity was classified into mild (FVIII > 5–30%), moderate (FVIII 1–5%), and severe (FVIII < 1%) according to remaining FVIII coagulant activity [11]. FVIII activity is expressed as percent of normal or as units/ml of plasma. Single unit of FVIII equal to the amount of FVIII in each ml of pooled, fresh, normal human plasma. Also, single FVIII unit/ml is 100% of normal [12].

### Methods

Whole blood samples (4-5 ml) were withdrawn in tri-sodium-citrate tubes for performing; complete blood count, coagulation profile (aPTT, PT, bleeding time), FVIII coagulant activity assay, FVIII inhibitor status by Bethesda assay, and LD-PCR for Inv22 detection.

#### FVIII inhibitor assay

FVIII is incubated with test plasma, FVIII was progressively neutralized over time, the status of inhibitor was determined according to how much of added standardized FVIII was neutralized and is expressed as Bethesda unit [11].

#### Detection of FVIII Inv22 gene

Genomic DNA was extracted from whole blood using the commercially available Gene Jet whole blood Genomic DNA Mini Kit (Molecular biology, Thermo Fisher Scientific, #K 0781, #K 0782, USA). LD-PCR for detection of Inv22 mutation of FVIII gene was done as described by Liu and colleagues [13]. We used four primers P, Q, A, B (iNtRON Biotechnology, Seongnam-si, Gyeonggi-do, 462–120, Korea). The PCR reaction mixture was prepared from LD-PCR kit (enzynomics Biotechnology, #P225A, Korea).

## Results

One-hundred-twenty hemophilic patients fulfilling the study inclusion criteria were enrolled in the current work.

Sixty percent (72 patients) of them had severe disease while, the remaining 40% (48 patients) were moderately affected. Mildly affected patients and recently treated with FVIII were excluded.

Our entire patients’ cohort were males, their ages ranged from 1.5–22 years old with mean age of 13.5 ± 5.6 and median age of 14.2 years.

They all have regular follow at our hemophilia clinics. They were treated with either plasma derived FVIII or recombinant FVIII concentrates and occasionally cryoprecipitate as “on demand” regimen only.

All recruited patients were evaluated for FVIII gene mutation, namely Inv22; positive results were recorded in twenty-seven cases (27/120, 23%). Severely affected patients displayed significantly higher frequency (33%) for the Inv22 mutation as compared with moderately affected peers (6%) as presented in Table 1.

**Table 1:** The prevalence of Interon22 inversion mutation identified in severe and moderate hemophilia patients

Degree	Severe HA N (%)	Moderate HA N (%)	Total N (%)	test	***P*** value
Mutation					
**Inv22 positive**				**12.05**	0.0005***
**N (%)**	24 (33)	3(6)	27 (23)		
**Inv22 negative** **N (%)**	48 (67)	45(94)	93 (77)		
**Total**	72	48	120		

Inv22intron22 inversion, HA hemophilia A, N number, % percent, ***highly significant, test chi-square

FVIII inhibitors were detected much more frequently in severe hemophilia patients (16/72, 22%) as compared to moderately affected peers (5/48, 10%), but the difference in proportion did not reach statistical significance as shown in Table 2.

**Table 2:** Prevalence of FVIII inhibitors between Severe and Moderate Hemophilia Patients

Inhibitors	F VIII inhibitor positive N (%)	F VIII inhibitor negative N (%)	total
Degree			
**Severe HA** **N (%)**	16 (22)	56 (78)	72
**Moderate HA** **N (%)**	5 (10)	43 (90)	48
**Total**	21	99	120
**Test**	**2.62**		
**P value**	**0.105**		

*HA* hemophilia A, *N* number, *%* percent, ***highly significant, test chi-square

**Fig. 1:**
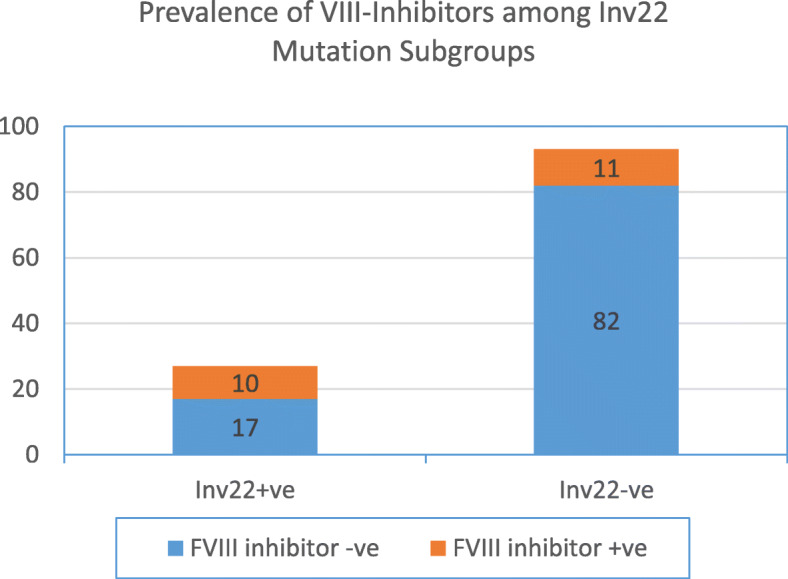
Prevalence of VIII-Inhibitors among Inv22 Mutation Subgroups

Regardless of disease severity, twenty-one (21/120, 18%) of all hemophilia patients developed inhibitors against FVIII. Those harboring Inv22 mutation showed significantly higher inhibitors proportion (37% (10/27) in their sera as compared to only 12% (11/93) among mutation- negative subgroup (X^2^ 8.9, *P* = 0.002). The risk of an inhibitor development during replacement therapy was four folds higher among Inv22 positive cases as compared with mutation negative peers (OR 4.3, 95% CI 1.6–11.9, *P* = 0.003). as displayed in Table 3 and Fig. 1.

**Table 3:** The Prevalence of FVIII Inhibitors among Interon22 inversion Positive and Negative Mutation Harboring Cases

Inhibitors	F VIII inhibitor positive N (%)	F VIII inhibitor negative N (%)	total
Mutation			
**Inv22 positive** **N (%)**	10 (37)	17 (63)	27
**Inv22 negative** **N (%)**	11 (12)	82 (88)	93
**Total**	21 (18)	99 (82)	120
**Chi-square**	**8.9**		
**P value**	0.002***		
**ODD Ratio (OR)**	4.3		
**95% CI**	1.6–11.9		
**P value**	0.003***		

Inv22 intron22 inversion, F VIII factor VIII, N number, % percent, ***highly significant,

In patients with severe hemophilia, Inv22 mutation significantly affected the development of FVIII inhibitor. Nine (38%) out of the 24 patients with severe diseases and Inv22 mutation-positivity, developed inhibitors while receiving replacement therapy, compared to only 7 (15%) out of the 48 patients with severe disease but with negative Inv22 mutation. The risk of inhibitor development was more than three folds increase when mutation is positive as compared with mutation-negative peers (OR 3.3, 95%CI 1.11–11, *P* = 0.03).

Similar findings were observed among moderately affected subgroup, as mutation-positive patients apparently, had 5 times increased risk of inhibitor development in their sera as compared to mutation-negative patients, but the difference in risk didn’t reach statistical significant level (OR 5.1, 95% CI 0.37–9.7, *P* = 0.2), as described in Table 4.

**Table 4:** Prevalence of FVIII inhibitors Among Severely and Moderately Hemophilia Patients with Different Interon 22 inversion Mutation Status

Inhibitor	F VIII inhibitor positive N (%)	F VIII inhibitor negative N (%)	Total
Degree Mutation			
**Severe HA**			72
**Inv22 positive N (%)**	9 (38)	15 (62)	24
**Inv22 negative N (%)**	7 (15)	41 (85)	48
**Odd Ratio**	**3.5**		
**95% CI**	**1.11–11**		
***P*** **value**	0.032*		
**Moderate HA**			48
**Inv22 positive N (%)**	1 (33)	2 (67)	3
**Inv22 negative N (%)**	4 (9)	41 (91)	45
**Odd Ratio**	**5.1**		
**95% CI**	**0.37–9.7**		
***P*** **value**	**0.2 #**		

Inv22 intron22 inversion, F VIII factor VIII, HA hemophilia A, N number, % percent, * significant, # not significant

It is of importance to mention that since patients were receiving treatment according to the type of the treatment modality available at the time of infusion, almost all our patients received nearly equal number of plasma driven and recombinant factor VIII infusions. Accordingly, we couldn’t draw correlations between the type of factor VIII infused on one hand and risk of inhibitors development and Inv22 positivity on the other hand. This was considered one of the limitations of our study.

## Discussion

Knowledge about the causative genetic defects of HA is warranted, for proper carrier detection and personalization of treatment plans according to underling mutation [14]. Assessment of prevalence of Inv22 mutation of FVIII gene among Egyptian hemophilia patients, and its role in FVIII inhibitor development was our main scope. Only severe and moderately affected patients were enrolled in the current cross-sectional study. Mild hemophilic patients were excluded as the prevalence of the target mutation; Inv22; among mildly affected patients is very low or even nil [15].

Inv22 mutation was detected in 23% of our cohort which is much less than that reported in related studies from different western and eastern countries [12, 14, 16–22]. This discrepancy can be explained by that most of the aforementioned researchers evaluated only severe hemophilia patients while we evaluated both moderate and severe patients. If moderate patients were excluded from estimation, our prevalence rate in severe cases only, approaches that recorded globally (33%).

Pan and colleague reported that Inv22 mutation caused 40–50% of severe hemophilia cases worldwide [23] while 17.5 to 46.4% frequency was recorded among Taiwanese hemophilia population [24]. The wide variation of Inv22 mutation prevalence in different studies (34–50%) [18] is probably due to different genetic distribution pattern in different ethnicities [12], and in part, due to different methods of its detection with variable sensitivities and specificities.

In general, Inv22 mutation was described as the causative genetic error of almost half of severe hemophilia cases [18]. The association between Inv22 mutation of FVIII gene and severe illness can be explained by its interference with the formation of full-length mRNA transcript leading to truncated and ineffective FVIII protein production and subsequently severe hemophilia [25].

Until recently, Inv22 mutation was not evaluated in developing countries due to many obstacles including the limited resources and difficulty of old techniques of its detection. The newer PCR techniques SPCR, LD-PCR [12, 26] rendered its recognition rapid and easy.

Development of antibodies against FVIII is the most distressing complication of FVIII replacement therapy, for both patients and treating physicians. These inhibitors destroy not only the infused factor, but also the small percentage of naturally synthesized factor by the body [9]. Consequently, these patients experience higher level of morbidity and mortality, and a worse quality of life due to poor orthopedic status [27].

Unfortunately, 18% of hemophilia patients enrolled in the current work were documented to have FVIII antibodies in their sera. This rate is in concordance with that reported from different studies [7, 16], but much higher than rates recorded in Pakistani (4%) [12] and Indian (8%) [28] studies.

Our results proved that, Inv22 mutation positive patients had significantly higher inhibitor prevalence rate than mutation negative peers (37% Vs 12%, *P* = 0.002). The risk of inhibitor development was significantly several folds higher among those affected by Inv22 mutation as compared with non-affected patients (OR 4.3, 95% CI 1.6–11.9, *P* = 0.003). This finding was documented by several authors [6, 16, 29]. Inv22 accounted for 33.3% of 533 patients with Hemophilia and 21% of them had inhibitors [7], 27.3–42.2% inhibitor rates were reported for Inv22 patient from smaller samples studies [30]. A 7–10 folds risk of treatment failure due to inhibitors was described in mutation bearing patients as compared to Inv22-negative correspondence. Conversely, the role of Inv22 as a risk factor for inhibitor development was denied by others [12].

Marked variability of inhibitors development rates (21–88%) [6] agrees with the conclusion that, antibodies formation is the outcome of interaction between several genetic and environmental factors [31]. Type of mutation especially Inv22, as declared from our results and many previous studies, represented an important genetic risk factor for inhibitors development. Other factors as race, MCH class, regimen of FVIII replacement, immunologic stress such as viral infection and immunization have been involved in inhibitor development [6–8].

Inhibitors were more frequently detected from sera of severely affected patients than from moderately involved subgroup of our cohort. Higher inhibitors rates among patients with severe disease was noted by many researchers [16, 32]. Severely affected patients usually suffer from significant bleeding tendencies with many bleeding episodes, and subsequently more exposure to FVIII replacement therapy. FVIII exposure especially intensive amount in relation to trauma and surgery was described as the most important relevant risk factor of inhibitor development [7]. This idea was supported with much lower inhibitor rates at India, where patients are very strictly treated on demand [28].

Severely diseased patients who harbor Inv22 mutation showed the highest prevalence rate of inhibitors than similarly affected patients but mutation negative. This finding supports the direct role of Inv22 mutation in inhibitor formation regardless of disease severity.

Kruse-Jarres in 2013, concluded that the plasma derived Von Willebrand factor containing may be less immunogenic than recombinant factor concentrates and may be suitable for those patients with severe disease or those at higher risk of inhibitors development [33]. We couldn’t assess this aspect in our cohort as most of our patients received almost equal infusions from both plasma driven and recombinant factor VIII.

The clinical implications of our study include; 1. In patients with Inv22 mutation positive, severe illness and increased risk of inhibitors development and treatment failure are expected. Hence, those patients need closer follow up and the situation should be explained thoroughly to the parents who should put more effort in taking care of their patients including trauma avoidance and perioperative precautions. 2. Recognition of the underlying causative mutation for hemophilia is important not only, to recognize the common mutation for each ethnicity and geographic region but also to determine patient’s prognosis. An information that enables treating physician to personalize treatment plan accordingly. 3. Patients harboring Inv22 mutation should be treated cautiously and strictly to minimize the chance of inhibitor development. Controlled early antigen presentation via prophylaxis seems promising, especially with strategies that avoid immunologic danger signals.

Our study had some limitation including; first, heterogeneity in regimen of replacement therapy as onset of first exposure, and its types; plasma, cryoprecipitate, factor concentrates, and recombinant factor with variable immunogenic responses [34] affecting inhibitor rates. Second, we couldn’t test the correlations of type and frequency of the infused factor VIII and inhibitors development and Inv22 mutation as patients almost received equal number of infusions from each type. Third, the cross-section nature of the study disabled accurate detection of onset of inhibitor where actual predisposing factors can be determined.

A multicenter randomized controlled trial is warranted to address those limitations. Questions about pathogenic mutation and its impact on disease course and management plan are still an area of research.

## Conclusions

Inv22 mutation is an important risk for severe illness and significantly predispose to FVIII inhibitor development. The prevalence of Inv22 of F VIII in Egyptian hemophiliacs is nearly like that of other population.

## Data Availability

All data and materials related to the study are included in the current manuscript.

## Abbreviations

HA: Hemophilia A
FVIII: Factor VIII
Inv22: Intron 22 inversion
aPTT: Partial thromboplastin time
PT: Prothrombin time
